# Influence of Orthodontic Anchor Screw Anchorage Method on the Stability of Artificial Bone: An In Vitro Study

**DOI:** 10.3390/ma13143205

**Published:** 2020-07-18

**Authors:** Seen-Young Kang, Ji-Min Yu, Hyoung-Sik Kim, Jun-Seok Lee, Chan-Mi Yeon, Ki-Sook Park, Sung-Hwan Choi, Seung-Youl Lee

**Affiliations:** 1 Medical Device Research Division, National Institute of Food and Drug Safety Evaluation, Chungcheong buk-do, Cheongju-si 28159, Korea; seenyoung@korea.kr (S.-Y.K.); yjm9223@korea.kr (J.-M.Y.); khs15ya@korea.kr (H.-S.K.); junseok1025@korea.kr (J.-S.L.); ycmi0331@korea.kr (C.-M.Y.); kispark@korea.kr (K.-S.P.); 2 Department of Orthodontics, Institute of Craniofacial Deformity, Yonsei University College of Dentistry, Seoul 02841, Korea; SELFEXAM@yuhs.ac

**Keywords:** orthodontic screw, titanium mini-implant, torque, artificial bone

## Abstract

This study aims to compare the torque values for various lengths of the titanium-based orthodontic anchor screw (OAS), different anchorage methods and varying artificial bone densities after predrilling. Furthermore, the effects of these parameters on bone stability are evaluated. A total of 144 OASs were prepared with a diameter of 1.6 mm and heights of 6, 8 and 10 mm. Artificial bones were selected according to their density, corresponding to Grades 50, 40 and 30. Torque values for the automatic device and manual anchorage methods exhibited a statistically significant difference for the same-sized OAS, according to the bone density of the artificial bones (*p* < 0.05). However, when insertion torque was at the maximum rotations, there was no significant difference in the torque values for the Grade 30 artificial bone (*p* > 0.05). When the torque values of both anchorage methods were statistically compared with the mean difference for each group, the results of the manual anchorage method were significantly higher than those of the automatic device anchorage method (*p* < 0.05). A statistically significant difference was observed in the bone stability resulting from different OAS anchorage methods and artificial bone lengths. These findings suggest that the automatic anchorage method should be used when fixing the OAS.

## 1. Introduction

Effective temporary anchorage is crucial for ensuring esthetic dentition and functional occlusion during orthodontic treatment [1,2]. Existing methods used for securing temporary anchorage include external anchorage devices such as head gear as well as internal anchorage devices such as Nance holding arches and trans palatal arches. However, orthodontic treatment with such devices requires patient cooperation; therefore, it is difficult to secure an adequate anchorage source [3,4]. Recently, orthodontic anchor screws (OASs) have been proposed as a reliable anchorage source because they do not require patient cooperation [3,4,5,6]. This method simplifies orthodontic treatment, has a wide range of oral applications, and allows for easy insertion and removal. Therefore, OASs have enabled orthodontic treatment in several cases in which it was previously impossible.

Initial stability is an important concern in the implementation of OAS procedures [7,8]. Several factors, including bone density, OAS diameter, OAS length and anchorage method, affect torque during the OAS procedure [9,10,11]. Specifically, bone stability affects the success and failure rate via the insertion torque [7,12,13]. In addition, bone density is related to the stability and retention of OASs [14,15,16]. If the complete anchorage is not achieved, micromovement may occur during initial healing, which can affect the failure rate and increase the potential for root and nerve damage [14,15]. Stability is affected by the diameter of the OAS, which typically ranges from 1.5 to 2.0 mm [16,17,18]; the larger the diameter, the greater the stability of bone implantation [19]. If the diameter is less than one millimeter, the OAS may exhibit instability because of micromovement [17]. Furthermore, the longer the OAS, the higher the holding force [19,20]. However, if the OAS is considerably long, the torque value will be large owing to invasion during deep placement in the oral cavity [19,20]. Therefore, the most stable OAS lengths are 6, 8 and 10 mm [19,20,21].

One of the key factors affecting torque during OAS placement is the type of the anchorage procedure used. OAS implantation methods are typically classified into automatic and manual methods [20,22]. Traditionally, the manual anchorage method involves predrilling the OAS into the bone, and then tightening it with a dedicated screwdriver. In contrast, the automatic device anchorage method employs predrilling the bone and using an automatic screwdriver to place the OAS at a constant force and speed [22]. Manual anchorage likely results in a higher insertion torque in the bone due to difficulty in maintaining a constant rotational speed or vertical force [23,24], while recent developments in automation equipment have achieved stable insertion at a constant speed and force [25].

Recently, OASs have been widely used in orthodontic treatments. However, few studies have investigated the effects of OAS anchorage method on bone stability, and few studies have compared the mechanical characteristics of the OAS length in the bone and the amount of torque produced by different OAS anchorage methods. Therefore, this study aims to analyze the effects of the OAS length, artificial bone density and anchorage method on bone stability by comparing torque values generated during anchorage. The null hypothesis of this study is that there will be no difference in torque values for different anchorage methods, OAS lengths or bone densities.

## 2. Materials and Methods

### 2.1. Study Design

**Figure 1:**
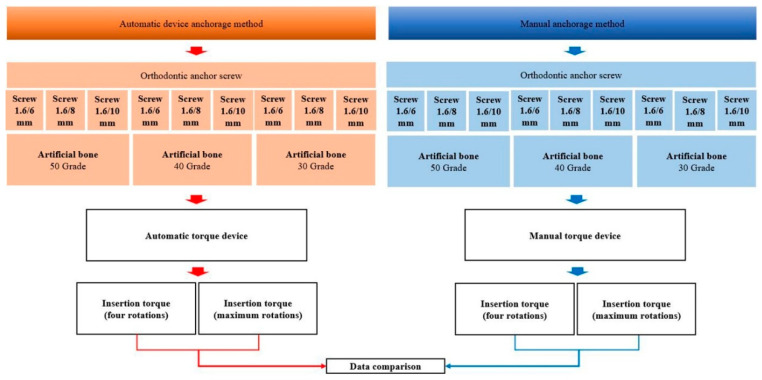
Overview of the experimental process.

The flow chart of the study protocol is shown in Figure 1.

**Figure 2:**
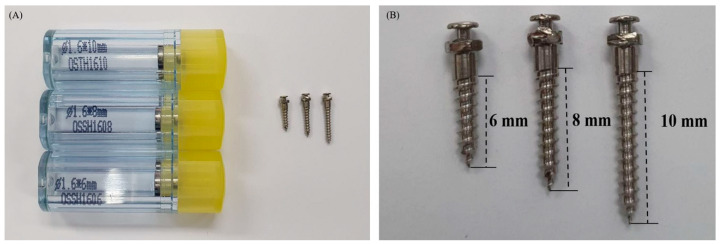
Orthodontic anchor screws used in the study. (**A**) Actual size of OAS; (**B**) Thread length of OAS.

**Figure 3:**
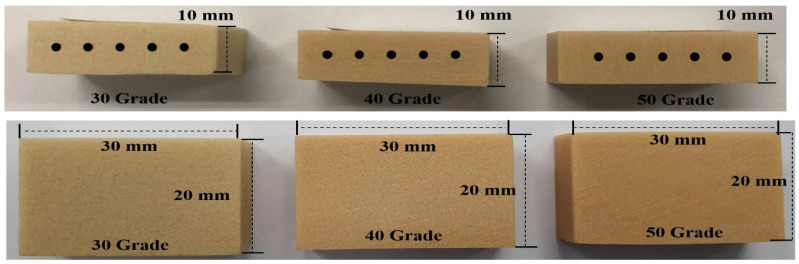
Different types of artificial bone used in the study.

In this study, 144 OASs (OSSH1606;1608;1610, Osstem Implant, Busan, Korea) were used with a uniform diameter of 1.6 mm and heights of 6, 8 and 10 mm (1.6/6 mm, 1.6/8 mm and 1.6/10 mm) (Figure 2). Artificial bones (Sawbone, Division of Pacific Research Laboratories, Inc., Vashon, WA USA) of Grades 50, 40 and 30 were selected according to their density, and then fabricated to a size of 10 × 30 × 20 mm (Figure 3). An OAS diameter of 1.6 mm was selected because this diameter value is the most stable between 1.5 and 2.0 mm [17]. The artificial bone used herein was made of polyurethane material, according to the ASTM F1839-08 standards. The density of the artificial bone was selected to coincide with the density of the jaw bone [26,27,28]; therefore, Grades 50, 40 and 30 bones were selected as their densities are closest to that of jaw bones [29].

**Figure 4:**
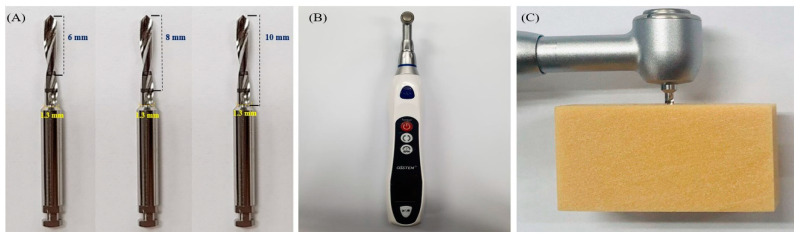
Predrilling of artificial bone. (**A**) orthodontic drills; (**B**) orthodontic automatic device; (**C**) predrilling method.

A dental drill device (E-driver, Osstem Implant, Seoul, Korea) was equipped with drills having a diameter of 1.3 mm and heights of 6, 8 and 10 mm (Ortho anchor, Osstem Implant, Seoul, Korea) (Figure 4). Then, predrilling was performed perpendicular to the artificial bone. Predrilling was conducted in the vertical directions of 6, 8 and 10 mm to the artificial bone depending on the length of the prepared OAS. Then, after the drill had entered the bone by approximately 2–3 mm, predrilling was performed only by rotational force and insertion speed was maintained at 20 rpm. The insertion torque was measured for each anchorage method.

### 2.2. Insertion Torque Measurement

#### 2.2.1. Insertion Torque Measurement Using Automatic Device Anchorage Method

**Figure 5:**
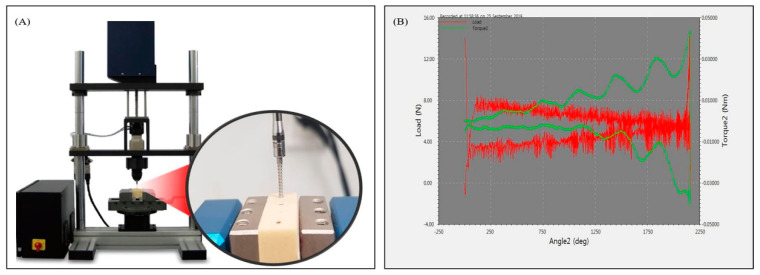
Insertion torque measurement using the automatic torque device. (**A**) automatic torque device (**B**) angle–torque graph.

An automatic torque device (Admet eXpert8600, ADMET, Norwood, WA, USA) (Figure 5A) was used for the automatic device anchorage method. The load-measurement and cross-head speed accuracy was ±0.5%. The driver tip of the device was fixed to the driver connection and the OAS was locked. The OAS was selected according to the depth of the pilot hole of the artificial bone. After fixing the artificial bone with a clamp and inserting the OAS in the predrilled position to ensure that the thread length of the OAS was approximately 60%, the maximum load was maintained at 1.14 kgf and the motor was moved to the head of the screw [30,31].

**Figure 6:**
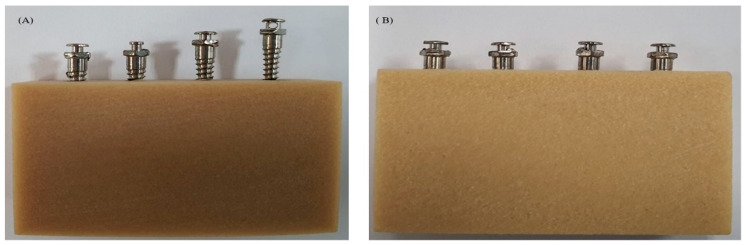
Insertion torque measurement. (**A**) four rotations (1440°) of OAS length, (**B**) maximum rotations of orthodontic anchor screw (OAS).

A torque of 3 rpm was applied clockwise with a torque wrench. When the OAS was inserted, the applied rotational speed and force were used to insert to 1 mm when the OAS rotated once. In the case of the automatic device anchorage method, the rotational speed and force of a screw were applied according to the international standards [30,31]. The insertion torque corresponding to four rotations was measured by applying a rotational force of 1440°; a maximum rotations was measured for each OAS length by applying rotations of 2160°, 2880° and 3600° for the 6-, 8- and 10-mm OASs, respectively (Figure 6). The measured value was set to the maximum torque record with the largest force in the screw rotation angle–torque graph using a software program (GaugeSafe software, ADMET, Norwood, WA, USA) (Figure 5B).

#### 2.2.2. Insertion Torque Measurement Using Manual Anchorage Method

**Figure 7:**
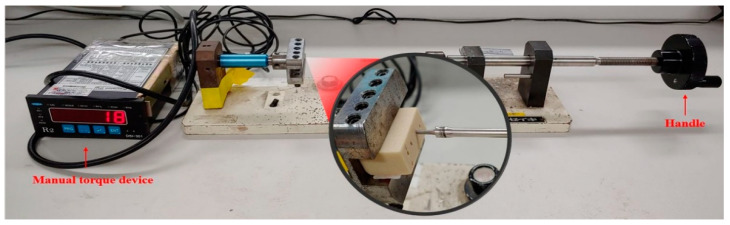
Insertion torque measurement using the manual torque device.

A manual torque device (DSI-301B, PARK ELECTRONICS, Busan, Korea) (Figure 7) was used for the manual anchorage method. The manual torque device facilitated measurement of the insertion torque by the connection of a DC strain gauge type sensor with an accuracy of 0.02%. The OAS was inserted into the artificial bone to convert the applied force, pressure, moment and weight into changes in electrical resistance to measure the insertion torque.

In the case of the manual torque device, the driver tip was fixed to the driver connection and locked with a hexagon wrench. After fixing the artificial bone on the bottom surface, the length of the OAS was locked at a predrilling position of 60%, and the torque was measured by manually applying a rotational force in the clockwise direction. In the manual method, the insertion torque was measured by turning the handle approximately 4, 6, 8 or 10 rotations, depending on the length of the OAS. When the OAS is inserted into the artificial bone and load is applied to a load cell, it is converted into a voltage change value correlating with the resistance change amount of the strain gauge. This voltage change is converted into N∙cm and recorded.

### 2.3. Statistical Analysis

Statistical analysis of the measurements was performed using a statistical software program (IBM SPSS Statistics v24.0, IBM Corp., Armonk, NY, USA). In reference to the bone density and OAS length, the insertion torque values were measured using the Kolmogorov–Smirnov test and Shapiro–Wilk test to determine normality. The nonparametric Kruskal–Wallis test was used to compare the mean values and analyze the significant differences. In addition, to identify significant differences between the automatic and manual anchorage methods, the nonparametric Mann–Whitney U Test was performed by applying a significance level adjusted by the Bonferroni method as a post hoc analysis.

## 3. Results

Table 1 lists the insertion torque results (four rotations) obtained by inserting OASs with different lengths into four artificial bones using the automatic device anchorage method. Statistically significant differences (*p* < 0.05) were observed in the insertion torque (for four rotations) for different bone densities; however, there was no statistically significant difference in the maximum torque (for four rotations) for different OAS lengths (*p* > 0.05).

Table 2 lists the results of the maximum rotational insertion torque of the OAS into various artificial bones using the automatic device anchorage method. Statistically significant differences (*p* < 0.05) were observed in the insertion torque (maximum rotations) for different bone densities. Moreover, most insertion torque values (maximum rotations) showed statistically significant differences (*p* < 0.05) for different OAS lengths; however, no significant difference was found for the Grade 30 artificial bone (*p* > 0.05).

Table 3 lists the insertion torques for the four rotations of the OAS into various artificial bones using the manual anchorage method. Statistically significant differences (*p* < 0.05) were observed in the insertion torque (four rotations) for different bone densities. Moreover, a statistically significant difference (*p* < 0.05) was observed in the insertion torque for different OAS lengths, except for the Grade 40 artificial bone (*p* > 0.05).

Table 4 lists the insertion torque values obtained by 100% insertion of the OAS into various artificial bones using the manual anchorage method. Statistically significant differences (*p* < 0.05) were observed in the insertion torque (at 100% insertion) for different bone densities. Moreover, a statistically significant difference (*p* < 0.05) was observed in the insertion torque for different OAS lengths, except for the Grade 30 artificial bone (*p* > 0.05).

Table 5 lists the torque values for four rotations for both the automatic and manual anchorage methods. For the same OAS lengths, the manual anchorage method exhibited a statistically higher torque than that exhibited by the automatic device anchorage method (*p* < 0.05). In addition, the overall average torque for Grades 50, 40 and 30 bone were 0.70, 1.29 and 1.37 N∙cm, respectively.

Table 6 compares the torque values at 100% insertion for the automatic and manual anchorage methods. The manual anchorage method exhibited a statistically higher torque than that exhibited by the automatic device anchorage method (*p *< 0.05). The overall average torque for Grades 50, 40 and 30 bone were 6.41, 3.74 and 3.95 N∙cm, respectively.

## 4. Discussion

The insertion torque is a key parameter because it is related to the holding force of the OAS and therefore affects the calibration performance [7,8,9]. The primary and secondary stability between the screw and bone is very important. primary stability is mechanical contact(friction) between the screw and bone [32], and secondary stability is remodeling and osseointegration of the bone between the screws [33]. However, after the orthodontic treatment, the secondary stability is meaningless because OAS must be removed from the jaw bone. Therefore, in this study, torque was measured for artificial bones of various bone densities, which were selected according to the various lengths of the OAS.

In addition, the densities of the artificial bone were selected to correspond to the densities of the jaw bones. Devlin et al. reported bone densities of 0.55, 0.31 and 1.11 g/cm^3^ for the anterior maxilla, posterior maxilla and mandible [25], respectively; Choel et al. reported the densities of the dental mandible and edentulous mandible to be 0.60 and 0.52 g/cm^3^ [26], respectively; and Kido et al. reported the densities of the anterior, premolar and molar regions to be 0.63, 0.57 and 0.52 g/cm^3^, respectively [27]. The artificial bone used in this study was made of a polyurethane material in accordance with the ASTM F1839-08 standard [28].

The automatic device anchorage method used in this study was a universal test that utilized a torque measuring device, which could quantitatively measure the constant speed and the amount of torque generated by the torque driver. The manual anchorage method was performed by using a device designed to measure the insertion torque via a constant rotating force.

In this study, the insertion torque was analyzed after predrilling the artificial bones of Grades 50, 40 and 30 artificial bone; this was aimed at objectively simulating and analyzing the insertion torque generated when the thread only rotates in the bone, according to the length of the OAS and the anchorage method.

In addition, it is meaningful to evaluate the torque values according to the OAS anchorage method. The International Standardization Organization test (ISO 19,023:2018 dentistry–orthodontic anchor screw) requires that the insertion torque be measured at four rotations; however, this study considered actual clinical conditions through measurement of the insertion torque in the artificial bone at four rotations and at maximum rotations.

Previous studies have employed various OAS stability values; however, the most suitable torque values for clinical applications are 5–10 and 15–20 N∙cm [11]. However, these torque values vary depending on the diameter of the OAS and the shape of the thread. It is difficult to determine a suitable value because the force is applied differently in the oral cavity. In addition, there are prior studies that refute this clinical value [29]. However, this study was conducted using the torque values in the range of 5–20 N∙cm to achieve an objective comparison by simulating the amount of torque generated in the artificial bone according to the length of the OAS and the anchorage method.

The results of this study showed that the artificial bone density, length of the OAS and anchorage method all had statistically significant (*p* < 0.05) effects on the artificial bones (Table 1, Table 2, Table 3, Table 4, Table 5 and Table 6). Specifically, by using the automatic device anchorage method, the insertion torque (four rotations and maximum rotations) increased with increasing bone density (*p *< 0.05). Moreover, the insertion torque maximum rotations increased with increasing OAS length (*p *< 0.05), except for Grade 30 bone, because the torque increases with the amount of contact and compression force between the bone and thread of the OAS due to an increase in the bone density and length of the artificial bone. However, the insertion torque at four rotations showed no significant effect on the OAS length (*p *> 0.05) because the same diameter and same number of rotations were employed for each OAS length during insertion. In this test, the torque was measured after inserting 60% of the OAS into the predrilled hole; thus, the torque value increased as the number of rotations increased, but did not change with an increase in the depth before rotation.

The insertion torque (maximum rotations) in the same artificial bone for various lengths of OASs in the predrilled hole increased as the number of threads increased, i.e., as the length of the OAS increased. This result coincides with previous studies [8,34,35,36,37,38]. However, this phenomenon was not observed in the Grade 30 artificial bone (Table 2). This is because an increase in the rotational speed of the OAS leads to an increase in the torque value, but this is not observed at a low bone density, which may indicate a problem in the OAS holding force. In this study, the Grade 30 bone density was approximately equivalent to that of the maxillary bone.

Some similar results were observed for the manual anchorage method, i.e., during the four rotations and the maximum rotations, the insertion torque increased as the bone density increased (*p* < 0.05). which coincides with previous experimental and simulative studies [27,34,36,38,39]. Furthermore, the insertion torque during the four rotations varied significantly with the OAS length, except for the Grade 40 bone. In contrast to the automatic device anchorage method, the manual anchorage method showed a statistically significant difference in the insertion torque (at all four rotations) with an increase in the OAS length because the force and the rotation speed of the operator were not constant [22,23,24]. Therefore, the success of the manual anchorage method is dependent on the skill of the dentist. Additionally, the insertion torque (maximum rotations) exhibited statistically significant variations with the OAS length, except for the Grade 30 bone. This trend was observed in both the manual and automatic device anchorage methods, indicating a problem in the initial stability during anchoring of the screw when the density of the jaw bone is similar to that of the Grade 30 artificial bone.

A clear difference was observed between the manual and automatic device anchorage methods with respect to the amount of torque in that the manual anchorage method typically exhibited a higher mean torque than that achieved for the automatic device anchorage method. Considering the clinical situation in the oral cavity, comparing the torque values according to the OAS anchorage method is difficult because multiple variables must be considered. In previous studies, however, the most appropriate torque values for the OAS placement were deemed to be 5–10 and 15–20 N∙cm [11]. If this value is judged as the clinical value, the bone density of Grade 30 is stable. However, when considering the length for achieving a stable anchorage of the OAS, long and short OASs are similar. Therefore, the bone thickness should also be considered in this case. When inserting an OAS with a diameter of 1.6 mm and a length of 10 mm into the Grade 50 bone by using the manual anchorage method, the insertion torque (maximum rotations) is more than 20 N∙cm. This value is outside the stable range and should always be considered carefully during anchorage. In the end, the automatic device anchorage method is thought to affect the initial stability because it reduces the insertion torque.

In previous studies, insertion torque was analyzed using artificial cortical and cancellous bones. The novelty of this study lies in the value of the non-predrilling insertion torque [40]. Moreover, when the insertion torque is measured without predrilling, the maximum rotations torque is inevitable in the cortical bone. However, in this study, the density of the artificial bone approximated the average density of the jaw bone, and the maximum rotations torque that could occur in the bone after predrilling was quantitatively quantified and measured. This is of great significance as it elucidates the degree of occurrence according to the procedure and length of the orthodontic anchorage device via the insertion torque generated in the bone, and it can be used as clinically and commercially quantifiable data.

In addition, the insertion torque data measured using actual jaw bones may differ from the results of this study [7,41]. Actual bone density is variable, so determining the objective value depending on the applied pressure is difficult. However, because the artificial bone has a specified density, it is easy to predict the numeric value generated according to the length of the screw or the anchorage method. Nevertheless, it can be difficult to evaluate whether the value is clinically appropriate.

According to the results of this study, many values of the parameters could be applied; however, considering the conditions, we make the following recommendations. First, the automatic device anchorage method should be used because the manual anchorage method results in a higher insertion torque. Second, a mandible with a density of greater than Grade 50 is expected to require automatic anchorage. Third, for a maxilla density of approximately Grade 30, the length of the OAS should be considered during the initial anchorage in the oral cavity because the retention force is similar even if the length of the OAS is increased.

This study represents an important contribution to the field of dentistry by evaluating torque values in the bone during OAS procedures. However, there are some limitations to this study. In clinic situation, the manual anchorage method is difficult to precisely control in vertical direction because the power, speed and technique are different depending on the dentist individual so that manual method is influenced by the skill of the dentist. In addition, it is difficult to analyze the insertion torque quantitively that occurs when the OAS is inserted into the jawbone. This is because in a clinical setting, even after the pilot hole is formed in the jawbone, the force and speed applied when the OAS is inserted increase, resulting in imbalance, and it is difficult to apply a constant vertical force. as the rotational resistance increases. The manual torque device used herein cannot maintain a constant force and speed as in the normal manual method; however, it can apply the force in a constant vertical direction when inserting the OAS and rotate the handle to see the result of the insertion torque. It provides a quantitative measurement of the torque. Therefore, the experiments in this study were conducted using a manual test device, and it is considered that the differences from the clinical situation can be described and analyzed. Moreover, this study used artificial bones, and the observed phenomena may differ in real bones, although, the selection of the most appropriate densities increased the validity of the test. Third, predrilling involved the construction of a hole at a fixed rotational speed at the best possible angle, and it is difficult to rule out error because the torque can vary with the diameter of the predrilling hole. To reduce this error, tests were conducted according to the instructions of the manufacturer to ensure that they are clinically meaningful. In future studies, an analytical tool should be developed to enable the operator to accurately measure the speed and force when using a manual torque measuring device. In addition, objective tests should be conducted using real jawbones to reflect actual clinical conditions.

## 5. Conclusions

Despite the limitations of this study, based on the finding of this in vitro study, we present the following conclusions:

1. The insertion torque increased as the length of the OAS increased; however, there was no statistically significant difference in the insertion torque in Grade 30 artificial bone (*p* > 0.05). This means that in the case of a jaw bone similar to the Grade 30 artificial bone, clinical failure may occur because of incomplete anchoring when the OAS is placed;
2. For a jaw bone having a density approximating that of the Grade 50 artificial bone, the automatic anchorage method is recommended when anchoring an OAS of 10 mm;
3. The automatic device anchorage method reduced the insertion torque of the OAS to a greater extent than the manual anchorage method;
4. Torque testing using artificial bones is clinically limited; therefore, future studies should be conducted using actual jaw bone of varying densities.

## Figures and Tables

**Table 1:** Insertion torque values (four rotations) measured using the automatic anchorage method.

Insertion Torque (Four Rotations)													
Thread Length of OAS	50 Grade				40 Grade				30 Grade				*p*-Value
	Mean/SD	MAX	MIN	MID	Mean/SD	MAX	MIN	MID	Mean/SD	MAX	MIN	MID	
**1.6/6 mm**	5.87 ± 0.35 ^a^	6	5	6	3.12 ± 0.35 ^a,b^	4	3	3	1.87 ± 0.35 ^b^	2	1	2	*p* < 0.05
**1.6/8 mm**	5.62 ± 1.18 ^a^	7	3	5	3 ± 0 ^a,b^	3	3	3	1.62 ± 0.51 ^b^	2	1	2	
**1.6/10 mm**	5.75 ± 0.46 ^a^	6	5	6	2.87 ± 0.35 ^a,b^	3	2	3	1.88 ± 0.33 ^b^	1	2	2	
***p*-value**	*p >* 0.05				*p >* 0.05				*p >* 0.05				

Unit—N∙cm; SD—standard deviation; MID—median; MAX—maximum; MIN—minimum; ^a,b^ values show statistically significant differences in the insertion torque between different artificial bones for the same OAS length (*p* < 0.05).

**Table 2:** Insertion torque values (maximum rotations) measured using the automatic device anchorage method.

Insertion Torque (Maximum Rotations)													
Thread Length of OAS	50 Grade				40 Grade				30 Grade				*p*-Value
	Mean/SD	MAX	MIN	MID	Mean/SD	MAX	MIN	MID	Mean/SD	MAX	MIN	MID	
**1.6/6 mm**	12 ± 2.50 ^a^	15	8	12	7 ± 1.06 ^a,b^	8	5	7	4.5 ± 0.92 ^a^	6	3	4.5	*p* < 0.05
**1.6/8 mm**	12.87 ± 2.69 ^a^	9	6	7	7.37 ± 0.91 ^a,b^	9	6	7	4.75 ± 0.88 ^a^	4	3	3.5	
**1.6/10 mm**	19.75 ± 3.80 ^a,^*	26	15	18	13.00 ± 1.60 ^a,b,^*	15	11	13	6.5 ± 1.41 ^a^	9	4	6.5	
***p*-Value**	*p <* 0.05				*p <* 0.05				*p >* 0.05				

Unit: N∙cm; SD—standard deviation; MID—median; MAX—maximum; MIN—minimum; ^a,b^ values show statistically significant differences in the insertion torque between different artificial bones for the same OAS length (*p* < 0.05); * values represent statistically significant differences in the insertion torque between different lengths of OAS for the same artificial bone density (*p* < 0.05).

**Table 3:** Insertion torque values (four rotations) measured using the manual anchorage method.

Insertion Torque (Four Rotations)													
Thread Length of OAS	50 Grade				40 Grade				30 Grade				*p*-Value
	Mean/SD	MAX	MIN	MID	Mean/SD	MAX	MIN	MID	Mean/SD	MAX	MIN	MID	
**1.6/6 mm**	7.12 ± 0.64 ^a,^*	8	6	7	4.62 ± 0.74 ^a,b^	6	4	4.5	4.00 ± 0.75 ^b,^*	5	3	4	*p* < 0.05
**1.6/8 mm**	5.62 ± 0.74 ^a^	7	5	5.5	4.12 ± 1.12 ^a,b^	6	3	4	2.62 ± 0.51^b^	3	2	3	
**1.6/10 mm**	6.62 ± 1.06 ^a^	8	5	6.5	4.12 ± 1.45 ^a,b^	7	3	3.5	2.62 ± 0.51^b^	3	2	3	
***p*-Value**	*p <* 0.05				*p >* 0.05				*p <* 0.05				

Unit: N∙cm; SD—standard deviation; MID—median; MAX—maximum; MIN—minimum; ^a,b^ values show statistically significant differences in the insertion torque between different artificial bones for the same OAS length (*p* < 0.05). * values represent statistically significant differences in the insertion torque between different lengths of OAS for the same artificial bone density (*p* < 0.05).

**Table 4:** Insertion torque values (maximum rotations) measured using the manual anchorage method.

Insertion Torque (Maximum Rotations)													
Thread Length of OAS	50 Grade				40 Grade				30 Grade				*p*-Value
	Mean/SD	MAX	MIN	MID	Mean/SD	MAX	MIN	MID	Mean/SD	MAX	MIN	MID	
**1.6/6 mm**	15.50 ± 1.19 ^a^	18	14	15	10.12 ± 1.72 ^a,b^	11	8	10.5	8.00 ± 1.51 ^a^	10	6	7.5	*p* < 0.05
**1.6/8 mm**	22.62 ± 1.59 ^a^	25	21	22	15.62 ± 2.06 ^a,b,^*	20	14	15	10.12 ± 1.72 ^a^	14	9	9.5	
**1.6/10 mm**	25.75 ± 2.49 ^a,^*	29	22	25.5	12.87 ± 2.23 ^a,b^	15	10	13.5	9.50 ± 1.41 ^a^	12	8	10	
***p*-Value**	*p <* 0.05				*p <* 0.05				*p >* 0.05				

Unit: N∙cm; SD—standard deviation; MID—median; MAX—maximum; MIN—minimum; ^a,b^ values show statistically significant differences in the insertion torque between different artificial bones for the same OAS length (*p* < 0.05); * values represent statistically significant differences in the insertion torque between different lengths of OAS for the same artificial bone density (*p* < 0.05).

**Table 5:** Comparison of insertion torque values (four rotations) between the automatic device and manual anchorage methods.

Insertion Torque (Four Rotations)												
Thread Length of OAS	50 Grade				40 Grade				30 Grade			
	MM	AM	MD	OA	MM	AM	MD	OA	MM	AM	MD	OA
**1.6/6 mm**	7.12 ^a^	5.87 ^b^	1.25	0.70	4.62 ^a^	3.12 ^b^	1.5	1.29	4.00 ^a^	1.87 ^b^	2.13	1.37
**1.6/8 mm**	5.62 ^a^	5.62 ^a^	0		4.12 ^a^	3 ^b^	1.12		2.62 ^a^	1.62 ^b^	1	
**1.6/10 mm**	6.62 ^a^	5.75 ^a^	0.87		4.12 ^a^	2.87 ^b^	1.25		2.62 ^a^	1.62 ^b^	1	

Unit: N∙cm; AM—automation method; MM—manual method; MD—mean difference; OA—overall average; ^a,b^ values represent statistically significant differences (*p* < 0.05).

**Table 6:** Comparison of insertion torque values (maximum rotations) between the automatic device and manual anchorage methods.

Insertion Torque (Maximum Rotations)												
Thread Length of OAS	50 Grade				40 Grade				30 Grade			
	MM	AM	MD	OA	MM	AM	MD	OA	MM	AM	MD	OA
**1.6/6 mm**	15.50 ^a^	12 ^b^	3.5	6.41	10.12 ^a^	7 ^b^	3.12	3.74	8.00 ^a^	4.5 ^b^	3.5	1.37
**1.6/8 mm**	22.62 ^a^	12.87 ^b^	9.75		15.62 ^a^	7.37 ^b^	8.25		10.12 ^a^	4.75 ^b^	5.37	
**1.6/10 mm**	25.75 ^a^	19.75 ^b^	6		12.87 ^a^	13.00	−0.13		9.50 ^a^	6.5 ^b^	3	

Unit: N∙cm; AM—automation method; MM—manual method; MD—mean difference; OA—overall average; ^a,b^ values represent statistically significant differences (*p* < 0.05).
